# Convergent genomic signatures of flight loss in birds suggest a switch of main fuel

**DOI:** 10.1038/s41467-019-10682-3

**Published:** 2019-06-21

**Authors:** Shengkai Pan, Yi Lin, Qiong Liu, Jinzhi Duan, Zhenzhen Lin, Yusong Wang, Xueli Wang, Sin Man Lam, Zhen Zou, Guanghou Shui, Yu Zhang, Zhengwang Zhang, Xiangjiang Zhan

**Affiliations:** 1 Key Laboratory of Animal Ecology and Conservation Biology, Institute of Zoology, Chinese Academy of Sciences, Beijing, 100101 China; 2 Cardiff University - Institute of Zoology Joint Laboratory for Biocomplexity Research, Beijing, 100101 China; 3 University of Chinese Academy of Sciences, Beijing, 100049 China; 4 Ministry of Education Key Laboratory for Biodiversity Sciences and Ecological Engineering, College of Life Sciences, Beijing Normal University, Beijing, 100875 China; 5 National Institute of Biological Sciences, Beijing, 102206 China; 6 State Key Laboratory of Integrated Management of Pest Insects and Rodents, Institute of Zoology, Chinese Academy of Sciences, Beijing, 100101 China; 7 State Key Laboratory of Molecular Developmental Biology, Institute of Genetics and Developmental Biology, Chinese Academy of Sciences, Beijing, 100101 China; 8 CAS Center for Excellence in Animal Evolution and Genetics, Chinese Academy of Sciences, Kunming, 650223 China

**Keywords:** Evolution, Evolutionary genetics

## Abstract

Flight loss in birds is as characteristic of the class Aves as flight itself. Although morphological and physiological differences are recognized in flight-degenerate bird species, their contributions to recurrent flight degeneration events across modern birds and underlying genetic mechanisms remain unclear. Here, in an analysis of 295 million nucleotides from 48 bird genomes, we identify two convergent sites causing amino acid changes in ATGL^Ser321Gly^ and ACOT7^Ala197Val^ in flight-degenerate birds, which to our knowledge have not previously been implicated in loss of flight. Functional assays suggest that Ser321Gly reduces lipid hydrolytic ability of ATGL, and Ala197Val enhances acyl-CoA hydrolytic activity of ACOT7. Modeling simulations suggest a switch of main energy sources from lipids to carbohydrates in flight-degenerate birds. Our results thus suggest that physiological convergence plays an important role in flight degeneration, and anatomical convergence often invoked may not.

## Abstract (web summary)

Flight loss has occurred numerous times in bird evolution. Here, the authors examine convergent sites in the exonic and intronic sequences of 48 bird genomes, finding amino-acid changes in two genes, *ATGL* and *ACOT7*, with potential implications for a change in metabolism rather than anatomy.

## Introduction

Feathered flight, an extremely energetically demanding form of locomotion^1^, is one of the most remarkable features of birds, distinguishing them from all other vertebrates. The origin of flight is generally accepted to lie in the avian ancestors (bird-like dinosaurs), based on evidence of avian characteristics of feathered dinosaur fossils^2^, such as feathers, wings, endothermic physiology and a unique pulmonary system with nine air sacs^3,4^. These earliest birds flourished on Earth around 150 million years ago (Mya), but are now all extinct. Modern birds, members of the crown group Neornithes arose in the Late Cretaceous^5^. Neornithes are classified into two subclasses: palaeognath and neognath^6^, which diverged 100 Mya^4^.

**Fig. 1:**
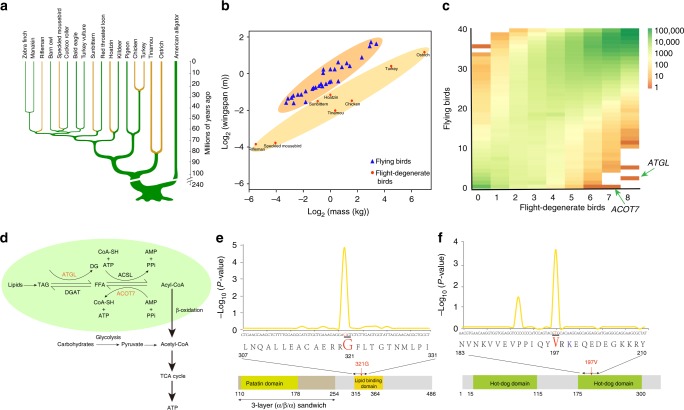
Identification of convergent evolutionary sites in flight-degenerate birds. **a** A phylogeny tree^5^ with American alligator as the outrgoup showing the convergence of eight studied flight-degenerate bird species. **b** Flying and flight-degenerate species were classified based on previous literatures and observations (Methods). Flight-degenerate species includes flightless birds and weak flyers. Average body mass of each species is plotted against its average wingspan measurement. **c** Two-dimensional frequency spectrum for nucleotides in flying and flight-degenerate species. The nucleotide frequencies of flight-degenerate birds were plotted against those of flying birds. The *x*-axis means the nucleotide counts among the eight flight-degenerate bird species and the *y*-axis means the nucleotide counts among 40 flying bird species. The number of loci is color-coded according to the scale on the right. **d** The pathway in which ATGL and ACOT7 are involved^35,70^. The main pathway for lipid metabolism is shadowed. In **e** and **f**, the upper shows the significant values (Fisher’s exact test) for the representative nucleotides on *ATGL* **e** and *ACOT7* **f**, the middle shows the sequence of amino acids, and the bottom shows the schematic domains. The identified convergent sites are highlighted in red. Source data are provided as a Source Data file

Because most extant birds have powered flight, it is inferred that the most recent common ancestor (MRCA) of all living birds had powered flight capability^7–9^, which however lacks genetic evidence. Strikingly, all palaeognaths and >60 species of neognaths have become flightless or weak flyers^10^ characterized by short burst of active and flapping flight (e.g. Galliformes and Tinamiformes^11^). This would mean that the degeneration of flight happened many times during the evolution of modern birds, from the older branches in the avian phylogenetic tree such as ostriches to recent branches such as riflemen (Fig. 1a). Recent studies claimed that loss of flight ability was due to multiple independent degeneration events during avian evolution^12,13^. A central question thus became: do these independent degeneration events of flight ability across more than 100 bird species have common underpinnings?

Previous studies have pointed out similar morphological changes in flight-degenerate bird species^14,15^. Darwin attributed the losses of flight to wing reduction^16^, and many subsequent efforts have been made to study structural modifications in flight-degenerate bird species. Recent molecular studies have identified particular coding or non-coding DNA sequences that may be involved in limb modifications in a single species (e.g. Galapagos cormorant^17^) or a single group (e.g. palaeognath^18^). Previous anatomical studies found that many flight-degenerate bird species (e.g. Galliformes^19^) differed from flying species in having a majority of fast glycolytic fibers in their flight muscles, functioning anaerobic metabolism of carbohydrates^20^, in contrast to flying bird species, which have mainly fast oxidative fibers capable of oxidative metabolism of both carbohydrates and lipids^20^. These, together with physiological observations such as higher lactose levels during running in several flight-degenerate bird species^21^, have resulted in the hypothesis that flight-degenerate bird species generally utilize carbohydrate as the main energy source when using their pectoral muscles for short burst of flight or running^22–24^, in contrast with use of lipid in flying species during sustained flight^25,26^. However, the genetic basis of such metabolic changes remains unknown.

In this study, we carry out a comparative genomic study of 48 avian species (40 flying and eight flight-degenerate) to explore convergent evolution of flight degeneration in modern birds. We identify convergent signatures occurring at two enzymes associated with lipid metabolism. Functional experiments show that these convergent substitutions significantly alter the enzyme activities in the flight-degenerate variants. Modeling simulations based on these functional changes predict a metabolic switch of main energy sources from lipids to carbohydrates in flight-degenerate birds. The ancestral reconstruction analysis finally enables us to reconstruct the history of flight degeneration during the 100 million years of evolution of modern birds.

## Results

### Genomic convergent signatures in flight-degenerate birds

To explore the genomic basis of the convergence of flight degeneration during the evolution of modern birds, we performed a comparative whole-genome analysis between eight flight-degenerate and 40 flying bird species^27^ (Fig. 1a, b). Our samples represent 92% of all extant avian orders (all of 32 orders in neognath and two of the five orders in palaeognath^27^). Initially we based our search on the expectation that phenotypic convergence can result from identical replacements of nucleotides occurring independently in unrelated taxa^28–30^. A convergent site in flight-degenerate bird species would be expected to be the same nucleotide shared among flight-degenerate bird genomes but distinct from the one dominant in flying species.

For the convergent evolution analysis, we first identified a total of 15,239 orthologous genes (including 8925 identified in previous work^27^ we contributed to and 6314 newly annotated using a reciprocal best-hit approach (Methods)). This is the most complete gene-set ever used in avian comparative genomic studies (e.g. 92% coverage of chicken genes). We calculated the frequency of each nucleotide for each SATé-aligned gene sequence^31^ among flying and flight-degenerate bird species in order to identify loci showing significantly different frequencies between flight-degenerate and flying species (*P* < 0.001, Fisher’s exact test; the nucleotide is shared by more than seven flight-degenerate species; Fig. 1c). Using this method, we scanned a total of 22,966,302 nucleotides (corresponding to 7,655,434 amino acids, AA) in exons and 272,215,340 nucleotides in introns, totaling about 25% of the whole avian genome on average. We identified 30 convergent candidates (29 genes), all of which were located in exons (Supplementary Table 1).

To eliminate potential false positives, we employed a recent method^32^ to check the 30 candidate nucleotides. The algorithm compares the observed convergent substitutions to those predicted from neutral expectation (Methods). A candidate is considered as a convergent signature only when its observed number is significantly higher than the neutrally expected one (*P* < 0.01, Poisson test). Only 8 of 30 candidate sites passed this strict criterion, and these were considered putatively convergent evolutionary sites for flight-degenerate avian species (Supplementary Table 1).

To further verify the reality of these eight convergent signatures, we increased the sample size to include all the 103 avian species (16 flight-degenerate vs 87 flying species) whose genome sequences are available in GenBank. The analysis of this bigger data set (Methods) confirmed only two of the eight sites that showed significantly different frequencies between flight-degenerate and flying bird species (*P* < 0.001, Fisher’s exact test) (Supplementary Table 2).

We localized the first site within the gene *ATGL* (*adipose triglyceride lipase*, site 961 in the chicken exon) and the second within *ACOT7* (*cytosolic acyl coenzyme A thioester hydrolase*, site 590 in the chicken exon). For *ATGL*, we found that the replacement of *A* by *G* on the convergent site results in an AA substitution in ATGL^321^ (Serine in flying bird species to Glycine in flight-degenerate bird species). For *ACOT7*, the convergent change (*C to T*) leads to an AA substitution in ACOT7^197^ (Alanine in flying species to Valine in flight-degenerate species).

A recent study of the flightless Galapagos cormorant^17^ identified no mutations in either *ATGL* or *ACOT7*. However, we note that these genes were missed from the genome they assembled. The study on Galapagos cormorants suggested that *CUX1* was an important candidate gene for the loss of flight, and could be associated with shorter wings. However, we found no nucleotide in this gene showing significantly different frequency between flight-degenerate and flying avian species in our samples (Supplementary Fig. 1).

### Functional significance of ATGL and ACOT7 substitutions

Both *ATGL* and *ACOT7* genes are involved in lipid metabolism (Fig. 1d). ATGL is one of the three main lipases expressed in multiple tissues in the body, as reported from previous physiological studies (e.g. adipose^33^ and muscle tissue^34^). It catalyzes the hydrolysis of triglyceride (TAG), the main form of energy storage in animals, and generates free fatty acids (FFAs) (Fig. 1d). Previous work showed that FFAs are further catalyzed by acyl-CoA synthetase to form acyl-CoAs^35^, which are processed by β-oxidation, with the products subsequently being used for energy generation through the tricarboxylic acid (TCA) cycle (Fig. 1d). ACOT7 counteracts this process through hydrolyzing acyl-CoA back to FFAs and CoA^35^ (Fig. 1d).

Therefore, either the reduced TAG hydrolytic activity of ATGL or the increased acyl-CoA hydrolytic activity of ACOT7 will, theoretically, decrease the formation of acyl-CoA, and reduce the generation of energy through lipid oxidation. Interestingly, the convergent site in ATGL is exactly located in the lipid-binding region based on its published 3D structure^36^ (Fig. 1e), and the convergent site in ACOT7 lies in the second thioesterase domain, which has an important role in the acyl-CoA hydrolytic activity^37^ (Fig. 1f). Given that pectoral muscles of flight-degenerate species typically possess extremely low proportion of oxidative fibers that can utilize lipid fuels^19,20^, we hypothesize that: (1) the AA substitution from Ser (flying species) to Gly (flight-degenerate species) at the convergent site of ATGL causes reduced lipid hydrolytic activity in flight-degenerate bird species, leading to less FFA production and thus less acyl-CoA and energy generation through lipid oxidation; and (2) the AA substitution from Ala (flying bird species) to Val (flight-degenerate bird species) at the convergent ACOT7 site enhances its activity in flight-degenerate bird species, hydrolyzing more acyl-CoA and thus lessening β-oxidation and its lipid energy generation.

**Fig. 2:**
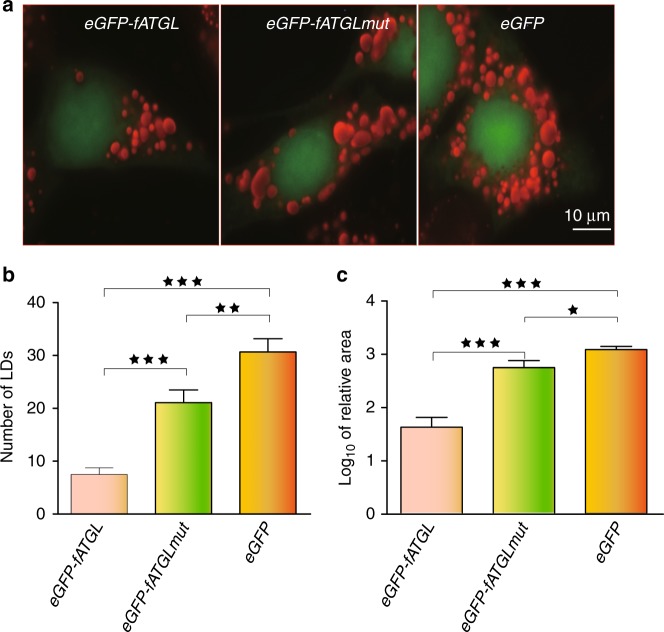
Functional validation of *ATGL*^*AGT321GGT*^ substitution. **a** The images were taken from the ORO stained human HeLa cells after the fixation with 3.7% formaldehyde: the wild type (*eGFP-fATGL*), mutant type (*eGFP-fATGLmut*) and negative control (*eGFP*). Lipid droplet numbers **b** and areas **c** were compared between the wild and mutant types (*N* = 90). *P < 0.05, **P < 0.01, ***P < 0.001 by t test. Error bars indicate s.e.m. Source data are provided as a Source Data file

To test that the amino acid difference in AGTL between flight-degenerate and flying avian species affects energy output from lipids, we carried out in vitro cellular experiments to compare the hydrolytic activity between ATGL^321Ser^ (flying species) and ATGL^321Gly^ (flight-degenerate species). We used the *ATGL* cDNA from a flying species (zebra finch) as the wild type (*fATGL*). Then we obtained the mutant type, *fATGL-mut* by mutating *AGT* to *GGT* at the 321st codon of *fATGL*, and used it to represent flight-degenerate bird species isoform. Each of the two complete (GFP-fused) cDNA sequences was cloned into the *pEGFP-N1* vector separately to express *fATGL* and *fATGL-mut* (Methods). GFP fluorescence detected in the transfected human HeLa cells and Western blotting verified the successful transfection and expression of *ATGL* (Fig. 2a and Supplementary Fig. 2). After 17 h’s incubation with 400 μM oleic acid (OA), we fixed the cells and used Oil Red O to stain unhydrolyzed OA to assess the lipid hydrolytic activity of ATGL. For each *ATGL* type, we randomly selected 90 GFP-positive cells and counted the number of lipid droplets (LD), the main form of unhydrolyzed OA, and we also measured the LD area in a cell. We found that the average number of unhydrolyzed droplets in the mutant type (i.e. flight-degenerate bird species) is three times higher than those found in the wild type (*P* = 5.0*E*-4, t test, Fig. 2a, b), and that the mutant has significantly larger area of lipid droplets than the wild (*P* < 0.01, t test, Fig. 2c). This implies that the amino acid substitution Ser321Gly in ATGL significantly reduces the enzyme’s hydrolytic activity on lipids.

**Fig. 3:**
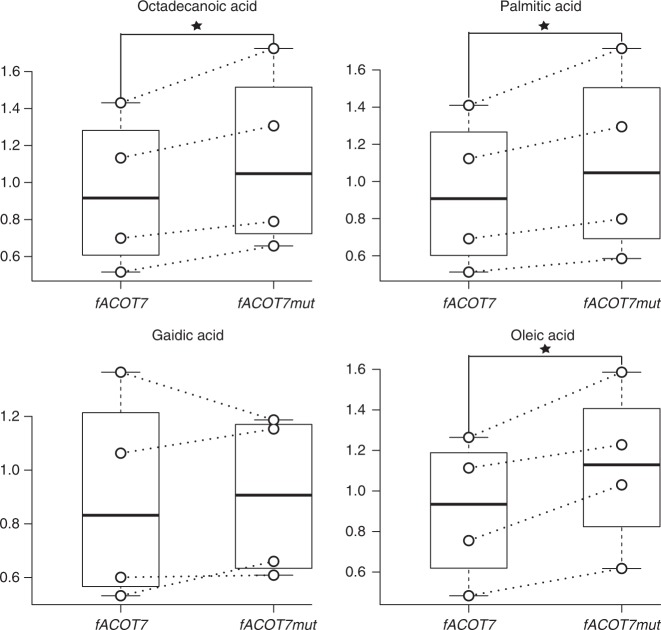
Functional validation of *ACOT7*^*GCG197GTG*^ substitution. The abundances of free fatty acids were measured using GC–MS in the mouse adipocyte cells expressed with *ACOT7* wild type (*fACOT7*) and *ACOT7* mutant type (*fACOT7mut*), respectively. For each free fatty acid, the abundance was compared between wild and mutant types and the median, first and third quartiles were shown in the box (middle bar represents median, upper bound the third quartile, lower bound the first quartile). Four independent experiments were performed. *P < 0.05 by the paired t test. Source data are provided as a Source Data file

To test that the difference of amino acid in ACOT7 also affects lipid-dependent energy output, we used *ACOT7* of the zebra finch (*fACOT7*) as the wild type and obtained mutant type (*fACOT7-mut*) through mutating *GCG* to *GTG* at the 197th codon (flight-degenerate bird species isoform). We cloned each of the two complete cDNA sequences into the viral vector *pCDH-CMV-MCS-EF1-copGFP* (Methods). Both the recombinant and helper plasmids were co-transfected into human embryonic kidney 293FT cells to collect virus containing target genes after 48 h. The virus was used to infect mouse 3T3-L1 preadipocytes. The adipocytes were collected to assess the hydrolytic activity for each *ACOT7* type after ten days of maturation. Because FFAs are the products of ACOT7 hydroxylation (Fig. 1d), we qualitatively and quantitatively measured FFAs in the differentiated cells with nine replicates for each *ACOT7* type using coupled gas chromatography–mass spectrometry (GC–MS). Our results showed that, among the four most common types of FFAs examined, the mutant type has significantly higher abundance of three FFAs than those found in the wild type (*P* < 0.05, paired t test, Fig. 3). These functional results thus support our hypothesis that the amino acid substitution Ala197Val in ACOT7 of flight-degenerate bird species could enhance its acyl-CoA hydrolytic activity, and suppress the energy production from lipids.

### Concerted functional changes of ATGL and ACOT7

Our results suggest that the genetic changes in the two genes in flight-degenerate bird species are both expected to reduce energy production through lipid oxidation. What contributions could these gene changes make to flight degeneration?

To address this question, we first analyzed the expression levels of the two genes in pectoral muscle tissues of chickens (flight-degenerate species representative, *N* = 6) and zebra finches (flying species representative, *N* = 6) respectively, as this tissue generates more than 80% of the energy required for flight^38^. We found that *ATGL* was expressed at least twice as strongly as *ACOT7* within each of the two species: the mean *RPKM* (*Reads Per Kilobases per Million reads*) was 86 in ATGL in contrast to 30 in *ACOT7* for chickens; and it was 114 vs 18 for zebra finches. We then directly compared the extent of FFA changes between *ATGL* and *ACOT7* mutant types expressed in mouse 3T3-L1 preadipocytes (Methods), since FFAs are the common metabolic products for both genes (Fig. 1d). Compared to *ACOT7*, our metabolomics analysis showed that the change of FFAs induced by *ATGL* substitution is about 2.5 times higher (*P* < 0.05, Wilcoxon rank sum test). Taken together, both the transcriptomic and metabolomic results suggest that the nucleotide substitution in *ATGL* had larger effects on flight degeneration than that of *ACOT7*.

**Fig. 4:**
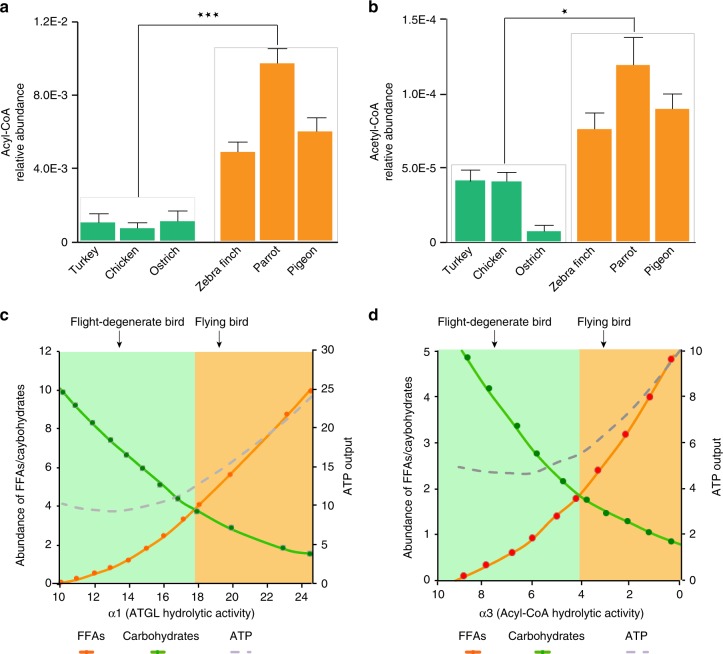
Modeling simulations of effects of functional changes on the energy landscape. The actual abundance of acyl-CoA **a** and acetyl-CoA **b** was measured in three flight-degenerate (turkey, chicken and ostrich) and three flying species (zebra finch, parrot and pigeon) using LC–MS. The simulated results for ATGL and ACOT7 are shown in **c** and **d** respectively. *P < 0.05, ***P < 0.001 by Wilcoxon rank sum test. Error bars indicate s.e.m. Source data are provided as a Source Data file

Next we wanted to know how the reduced lipid hydrolytic activity of ATGL coupled with the increased activity of ACOT7 reshapes the energy landscape in the pectoral muscles of flight-degenerate avian species. Energy production is normally a homeostatic process with lipids and carbohydrates serving as the two main resources, and lipid and carbohydrate metabolisms coordinately regulating energy homeostasis through negative feedbacks^39^. Basically, the inhibition of carbohydrate metabolism by lipid is mediated by the increase of mitochondrial ratio of [acetyl-CoA]/[CoA] triggered by fatty acid oxidation, which suppresses the activity of key glycolytic enzymes^40,41^. In our study, the concerted functional changes in ATGL and ACOT7 in flight-degenerate bird species are expected to cause a lower level of acyl-CoA, and consequently less acetyl-CoA production through fatty acid oxidation in flight-degenerate bird species. We have verified both these expectations by the metabolic comparisons between flight-degenerate and flying bird species: flight-degenerate bird species produce significantly less of both acyl-CoA and acetyl-CoA in their pectoral muscles than do flying bird species (both *P* < 0.05, Wilcoxon rank sum test, Fig. 4a, b). Furthermore, we found that there was no significant change of CoA concentration between the two bird groups. We therefore expected that there should be a lower mitochondrial [acetyl-CoA]/[CoA] in the pectoral muscles of flight-degenerate bird species. Based on these observations and conclusions, we predicted that the activity changes of the two key enzymes in flight-degenerate bird species will alleviate the suppression of lipid metabolism on carbohydrate metabolism, enabling flight-degenerate bird species to utilize more carbohydrates for energy production in their flight muscles. To test this prediction, we established a kinetic model mainly based on the antagonistic nature of these two pathways (details in Methods).

Our modeling simulations (*β*1 = *β*2 = 1, *n*1 = *n*2 = *n*3 = 1, *α*2 = 5) suggested that the diminished hydrolytic activity of lipases (e.g. decreasing *α*1 in Fig. 4c) or the increased hydrolytic activity of acyl-CoA (e.g. increasing *α*3 in Fig. 4d) not only reduces the energy generated from lipid metabolism, but promotes the alternative energy pathway of carbohydrate metabolism. When hydrolytic activity of the ATGL is reduced or ACOT7’s is increased to a threshold, the carbohydrates will become the dominant energy source (*α*1 = 18 or *α*3 = 4 in Methods, Fig. 4c, d). Our simulations thus suggest that flight-degenerate avian species will use carbohydrates as the major energy source, consistent with the findings of previous physiological studies in several flight-degenerate species (e.g. chicken^22^, turkey^23^, and ostrich^24^). Our simulations also predict that this turnover of the major energy source would result in lower energy generation in flight-degenerate species (Fig. 4c, d), which is confirmed by the observation of less acetyl-CoA in the pectoral muscles, the direct substrate for TCA cycle and ATP generation, in our metabolic analysis of three flight-degenerate species (Fig. 4b).

Energy to power pectoral muscles is the most important physiological prerequisite to enable bird flight^38^. Therefore, it was not unexpected that the change of dominant energy source should have an essential role in the evolution of flight degeneration. Based on our results, we propose the changed function of the pectoral muscles in flight-degenerate bird species is realized by the turnover of the main fuels from lipids to carbohydrates. Specifically, low amount of FFA is used for energy production in flight-degenerate bird species because of the sharp decrease of TAG hydrolytic activity caused by the substitution from Serine to Glycine in ATGL and the accompanied increase of acyl-CoA hydrolytic activity underlined by Alanine to Valine in ACOT7. Consequently, the carbohydrate utilization is increased due to negative feedback between lipid and carbohydrate metabolisms, with carbohydrates alternatively chosen as the main energy fuel. We thus provide the direct evidence of convergent evolution of metabolism in flight-degenerate bird species, providing mechanistic insights into the evolution of flight degeneration.

### Loss of flight is an adaptive trait

**Fig. 5:**
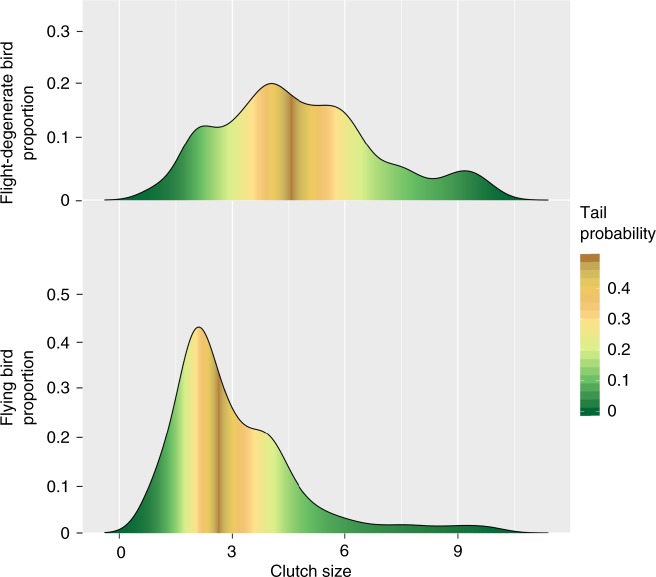
Comparison of clutch sizes between flying and flight-degenerate avian species. Clutch size data of 2036 flying and 226 flight-degenerate avian species from a previous study^49^ were analyzed. The *x*-axis means clutch size and the *y*-axis means the proportion of bird species. The color represents the cumulative tail probability of the distribution. Significance was assessed by a t test. Source data are provided as a Source Data file

PAML analysis for the two convergent loci showed both of them are subject to positive selection in flight-degenerate avian species (*P* = 0.970 for *ATGL* and 0.975 for *ACOT7*), supporting the previous hypothesis that the loss of flight is a result of natural selection^16,42^. We suggest two factors that could drive this selection: the need for fast escape response, and a higher reproductive rate. First, flight-degenerate bird species are flightless or weak flyers like Galliformes, and escape from predators by running or burst of flight in a quick response^11^. This unique locomotion style usually requires a rapid muscle contraction and high levels of force generation^43–47^. The fast carbohydrate-dependent glycolytic fibers^43–47^ are therefore the best option because they generate more and faster power per unit muscle mass than slow and fast oxidative fibers^20^. Second, our analysis of avian clutch size^48^ shows that flight-degenerate species (*N* = 226), on average, have significantly larger clutch sizes than flying species (*N* = 2036, *P* < 2*E*-16, t test, Fig. 5). The relatively lower energy requirement per individual may therefore confer a benefit for them through maximizing reproduction success^16^, permitting larger population sizes and greater probabilities of population survival^42,49,50^.

### Loss of flight during the evolution of modern birds

Our finding of two major causative genes associated with flight degeneration, together with the best understood phylogeny of modern birds^5^, allows us to reconstruct a continuous history of flight degeneration during the 100 million years of evolution of modern birds, and suggest an alternative to the generally accepted hypothesis that the most recent common ancestor of modern birds was a strong flyer.

**Fig. 6:**
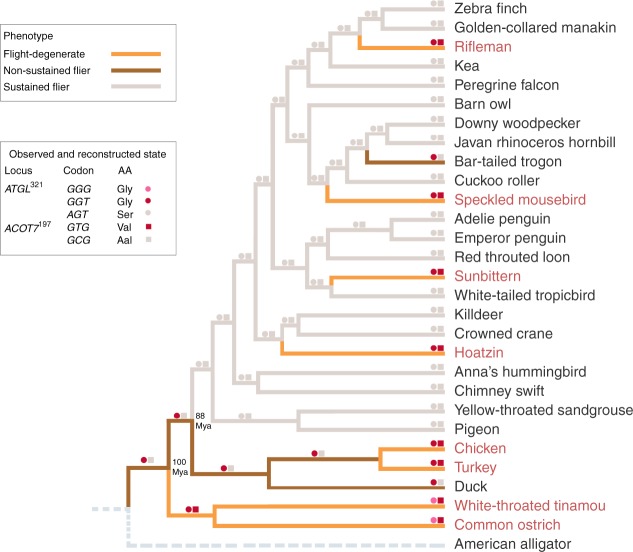
Ancestral state reconstruction for *ATGL*^321^ and *ACOT7*^197^ codons. The avian phylogeny from the previous study^5^ that we contributed to was used with the American alligator genome used as the outgroup

Using the American alligator (*Alligator mississippiensis*), one of the closest living relatives of modern birds, as the outgroup^51^, our reconstruction by PAML revealed that the most recent common ancestor of Neornithes that lived about 100 Mya^5^ are non-sustained flyers (i.e. *GGT* for the 321 codon of *ATGL* with posterior probability (*PP*) of 1.000; and *GCG* for the 197 codon of *ACOT7* with *PP* 1.000; Fig. 6), consistent with the previous claim^2,3^ but not others^52^. To the best of our knowledge, this is the first genomic work favoring the hypothesis that the Neornithes MRCA was a non-sustained flyer. This idea is supported by a recent neornithine ecological reconstruction work showing that ecological filtering due to the loss of significant plant cover across the Cretaceous-Paleogene boundary selected against flying dinosaurs (Avialae)^53^.

Our results imply that the non-sustained flight capacity was maintained in the first neognath bird (*GGT* for *ATGL*, *PP* = 1.000; *GCG* for *ACOT7*, *PP* = 0.967), and sustained flight ability occurred only in the Neoaves at about 88 Mya (*AGT* for *ATGL*, *PP* = 1.000; *GCG* for *ACOT7*, *PP* = 1.000; Fig. 6). It has long been unclear why Neoaves dominates modern birds; we suggest that strong flight ability, due to enhanced lipid metabolism, evolved in the Neoaves MRCA and allowed sustained flyers to take far more advantage of newly opened ecological niche space vacated by victims of the Cretaceous-Paleogene mass extinction about 66 Mya^52^, leading to their rapid radiation.

The ancestral codons of paleognath birds were inferred to be *GGG* for *ATGL* and *GTG* for *ACOT7* (both *PP*s = 1.000), consistent with the maintenance of these codons in all extant paleognaths, which are therefore inheritably flightless or weak-flighted (Fig. 6). Therefore, the flightlessness or weak flight ability is actually a synapomorphous trait among palaeoganths, contradicting the convergent evolution hypothesis usually invoked to explain evolution of flightlessness among ratites^15^. Moreover, our finding that tinamous share the *GGG* for *ATGL* and *GTG* for *ACOT7* suggests that this branch also maintained the ancestral flightlessness or weak flight ability (Fig. 6), which is in agreement with some earlier reports^10^ but not others^18^. Indeed, our analysis of the relationship between flight capacity (e.g. wingspan) and body weight suggests that the tinamou is more closely related to weakly flying bird species such as chickens, than to flying bird species (Fig. 1b).

Most importantly, our hypothesis explains very well how the moderately flying neognath ancestors evolved into the Galloanseres with the Galliformes birds losing their flight ability, and evolved into strongest flying Neoaves with some species such as hoatzins^10^, sunbitterns^54^, riflemen^10^ secondarily becoming flight-degenerate (Fig. 6). An interesting convergent selection is also found between the duck and bar-tailed trogon, which possess the genotypes of reduced *ATGL* (*GGT*) and lowered *ACOT7* hydrolytic ability (*GCG*), suggesting they are genetically capable of flying but in a non-sustained manner (Fig. 6), consistent with the behavioral observations^55^. It is also noted that our reconstruction results were confirmed using more taxa (*N* = 83) and different tree topologies (*N* = 17; Methods; Supplementary Fig. S4).

## Discussion

Some recent studies claim that there are no common and major-effect genes that determine the large-scale convergence across taxonomic orders^56^. However, previous work has not had the advantage of a well-sampled genome-based test. Utilizing one of the best developed avian genome databases so far^5,27^, we discovered that at least two major-effect genes under positive selection can explain the differences in flight styles during the 100 million years of evolution of modern birds. Obviously other processes, under the control of other genetic loci may also contribute to these spectacular evolutionary events (e.g. Supplementary Table 1).

For centuries, recurrent losses of flight in modern birds have been put forward as a classical example of functional degeneration that is predominately caused by structural reduction^16,17^. However, we found no shared gene sequence underpinnings for anatomical similarities in the flight-degenerate avian species (Supplementary Table 1). We cannot exclude the possibility of amino acid convergence without nucleotide convergence since the same amino acid could be coded by different codons in different flightless birds, which warrants further investigation. Besides, future genetic studies (e.g. gene gain and loss, structural variation, and gene regulation) will also be necessary to rule out the anatomical convergence as a main driver. Instead, we found that the loss of flight in modern birds is predictable with two key genes regulating carbohydrate and lipid metabolisms (Fig. 6). Our study thus suggests physiological convergence plays an essential role in a key behavioral transition, which involves a metabolic switch of the main source of energy between flight-degenerate and flying bird species. This can not only save energy^42^, but also be appropriate for flightlessness or short bursts of flying versus sustained flying.

## Methods

### Classification of flight-degenerate and flying species

We obtained the flight ability description for the 48 bird species from previous literatures^5,27,55,57^, which are used to group the studied avian species into two main groups: flying bird species and flight-degenerate bird species (Fig. 1b). Flight-degenerate bird species include two flightless bird species, ostrich (*Struthio camelus*) and turkey (*Meleagris gallopavo*), and six weak-flighted species, rifleman (*Acanthisitta chloris*), sunbittern (*Eurypyga helias*), speckled mousebird (*Colius striatus*) and hoatzin (*Ophisthocomus hoazin*), chicken (*Gallus gallus*) and white throated tinamou (*Tinamus guttatus*). Flying bird species are listed in Supplementary Table 2. It is noted that the two penguin species (*Aptenodytes forsteri* and *Pygoscelis adeliae*) were grouped into flying bird species because penguins have been described as *flying* in water and have a similar metabolic capacity to flying bird species^58^. We also obtained the data of wingspan and body mass from previous studies^59^ and checked their relationship in all studied bird species.

### Orthologous gene identification and alignment

The 8925 orthologous genes were obtained from the previous study^27^ we contributed to, in which we have sequenced, assembled and compared the full genomes of 48 bird species. In addition, we annotated another 6314 genes using a reciprocally best-hit approach. Briefly, with the chicken protein set used as a reference (Ensembl89), the protein set of each studied species was BLASTed against the reference and reciprocal best-match pairs were considered as orthologs. We performed multiple sequence alignment for each orthologous gene using SATé-II with default setting, which has been reported to be fast and accurate simultaneous estimation of multiple sequence alignments^31,60^.

Orthologous introns were identified only for those orthologous genes with more than two exons. For each orthologous gene, we first identified orthologous exons by Blasting each exon of chicken against the corresponding orthologous genes in the other 47 avian species. The fragment between two neighboring orthologous exons was determined as an orthologous intron. Multiple sequence alignment for each orthologous intron was performed using SATé-II with default setting.

### Identification of convergent signatures

We identified the convergent evolutionary sites in flight-degenerate avian species using the pipeline listed as below.

First, because previous research suggested that phenotypic convergence in unrelated taxa may be related to identical replacements of single nucleotides^28–30^, we expected that one feature for a site with convergent signal in flight-degenerate bird species is that the majority of flight-degenerate species share the same nucleotide, which however is distinct from the one dominant in flying bird species. Therefore, for each site in each gene, we calculated the frequency of a nucleotide dominating in flight-degenerate bird species (that is shared by at least seven flight-degenerate bird species) and its frequency in flying bird species. The frequency difference was examined by a Fisher’s exact test. The nucleotide shared by at least seven flight-degenerate bird species with the significance value <0.001 was considered as a convergent candidate.

Second, we implemented an approach published recently^32^ to confirm the convergent candidates identified in the first step. This method detects the convergent signal by testing whether the observed number of convergent substitutions in the studied gene among flight-degenerate bird species exceeds the expected (neutral) expectation. In the simulation, the avian phylogeny we used is obtained from the previous study^27^ and we analyzed the convergent signals among flight-degenerate bird species. Specifically, we inferred the ancestral amino acids at all internal nodes in the phylogeny, relative substitution rate for the considered site and all branch lengths of the phylogenetic tree by inputting the sequence alignment of candidate genes to the *codeml* program in PAML v4.7^61^, in which we used the parameters recommended by a previous study^32^: the *Empirical* + *F* model together with the *JTT-f*_*gene*_ matrix and a discrete gamma model with four rate categories. The branch lengths, relative substitution rate, and ancestral sequences inferred were then used to calculate the observed and expected numbers of convergent substitutions in this gene based on a probabilistic model of amino acid substitution^32^. Finally, Poisson cumulative-distribution function (*poisson.cdf* implemented in *scipy.stats* package in *Python*) was used to assess the significance of observed and expected numbers in the target gene.

Third, the convergent candidates identified in the second step were further verified in more avian species (*N* = 103) whose genome sequences are available in GenBank until 08/11/2018, of which 17 were classified as flight-degenerate bird species (Supplementary Table 2) again based on previous literature^54^. Basically, for each gene containing a convergent candidate, we first searched its orthologous gene in each of the 103 genomes using BLAST, and then aligned these orthologs using SATé-II^31^. For the nucleotide shared by at least 10 flight-degenerate bird species, its frequencies in flight-degenerate bird species and flying bird species were calculated followed by a Fisher’s exact test. The locus with *P* < 0.001 was considered as a convergent site.

### Functional assay of *ATGL*

The complete *ATGL* cDNA sequence of zebra finch (*ATGL*^*321AGT*^) was used as wild type and *ATGL*^*321GGT*^ as mutant type. The wild and mutant *ATGL* sequences were synthesized by TsingKe Biological Technology, cloned into *pEGFP-N1* vectors (Clonetech) separately, expressing ATGL with GFP at the *C*-terminus. The constructs were verified by DNA sequencing.

Human HeLa cells were cultured on glass coverslips with Dulbecco’s modified Eagle’s medium (DMEM) (Hyclone) supplemented with 10% FBS (Gibco) and 1% Penicillin- Streptomycin (P/S) (Gibco) (complete medium), and allowed to adhere overnight. The plasmid *pEGFP-N1* (negative control), and two recombinant plasmids were transfected into Human HeLa cells separately following the protocol of Lipofectamine 2000 (Invitrogen). After 6 h, the medium was replaced by the one with 400 uM oleate complexed to BSA (Sigma) (6:1 molar ratio), which facilates the formation of TAG-containing lipid droplets^62^. These were used as the substrates for ATGL lipase, and incubated for 17 h with subsequently replaced by complete medium. After another incubation for 24 h, glass coverslips were taken out and washed by PBS for three times. The cells were sequentially fixed by 3.7% formaldehyde solution for one hour, washed for three times using deionized water, and stained with ORO for 30 min in the dark. The stained cells were then subject to imaging on a fluorescence microscope (Nikon Eclipse 80i) under the 100X oil immersion objective. For each cell type, immunofluorescent images from 90 randomly selected positive cells were taken randomly, followed by the quantification of lipid droplet numbers and areas with ImageJ^63^. The mean comparison between different cell types was performed by a t test after the normality test in *R*.

The successful expression of transfected ATGL was confirmed by Western blotting using ATGL antibody (1:2000, Cell Signaling Technology, 2138 S) with α-Tubulin (1:10,000, Abcam, ab7291) as the internal control (Supplementary Fig. 2). The human HeLa cells were collected and lysed in the SDS-loading buffer. Protein lysates were separated by SDS–PAGE, and transferred to the NC membranes (GE). After blocking with 5% skim milk, the membranes were incubated with primary antibodies (ATGL antibody and α-Tubulin) overnight at 4 °C followed by the incubation of secondary antibodies (Goat anti-rabbit antibody (1:10,000, LI-COR Biosciences, 926–68071) for ATGL and Donkey anti-mouse antibody (1:10,000, LI-COR Biosciences, 926–32212) for α-Tubulin).

### Functional assay of *ACOT7*

The complete cDNA sequences of *ATGL* wild type, *fATGL* (*ATGT*^*321AGT*^), *ATGL* mutant type *fATGLmut* (*ATGT*^*321GGT*^), *ACOT7* wild type *fACOT7* (*ACOT7*^*197GCG*^), and *ACOT7* mutant type *fACOT7mut* (*ACOT7*^*197GTG*^) were synthesized by TsingKe Biological Technology, which were cloned into *pCDH-CMV-MCS-EF1-copGFP* vectors, respectively. The constructs were verified by DNA sequencing.

Human embryonic kidney 293FT cells were grown in DMEM supplemented with 10% FBS and 1% P/S. The *pCDH-fACOT7*, *pCDH-fACOT7mut*, *pCDH-fATGL*, *pCDH-fATGLmut*, and *pCDH* plasmids (negative control) were transfected into the cells with the helper plasmids (*psPAX2* and *pMD2.G*), respectively, using the Lipofectamine 2000 reagent. The medium was renewed after 6 h, and lentivirus was collected after 48 h, and filtered through a 0.45 µm filter (Millipore).

Mouse 3T3-L1 preadipocytes were grown in DMEM supplemented with 10% fetal calf serum (Gibco) and 1% P/S and incubated overnight. The medium was replaced by the fresh medium containing lentivirus supernatants and 10 µg/ml Polybrene (Millipore) and incubated for 12 h. Then, the medium was removed and replaced by DMEM supplemented with 10% fetal calf serum and 1% P/S. DMEM supplemented with 10% FBS, 1% P/S, 5 µg/ml Insulin (Sigma), 0.4 µg/ml Dexamethasone (Sigma), 0.5 mM Isobutylmethylxanthine (Sigma) was used to induce cell differentiation for two days until the grew cells covered the full culture dish (Day0 for differentiation). After another two days, the medium was replaced by DMEM supplemented with 10% FBS, 1% P/S, 5 µg/ml Insulin. Cells were subsequently re-fed with fresh DMEM supplemented with 10% FBS, 1% P/S every two days until Day10.

The maturation of mouse 3T3-L1 preadipocytes was determined by the RT-qPCR analysis of three genes (*CEBPβ*, *CEBPα*, *PPARγ*) relevant to adipocyte differentiation with two housekeeping genes *GAPDH* and *Cyclophilin A* as the internal control. Briefly, the matured adipocytes were collected and the total RNA was extracted using TRIzol (Invitrogen). Reverse transcription was carried out using GoScript Reverse Transcription System (Promega) to obtain cDNA. The qPCR was performed using the PowerUp SYBR Green Master Mix (ThermoFisher) and Agilent real-time PCR System (Agilent). The primers were derived from previous studies^64^ (Supplementary Table 3).

The successful expression of infected ACOT7 was confirmed by Western blotting (Supplementary Fig. 3). The differentiated mouse 3T3-L1 adipocytes were collected and lysed in the cold RIPA buffer (ThermoFisher) together with the protease inhibitors mixture. Protein lysates were separated by SDS–PAGE, and transferred to the PVDF membranes. After the block with 5% skim milk, the membranes were incubated with primary antibodies (ACOT7 rabbit polyclonal antibody (1:1000, Proteintech, 15972-1-AP) and β-Actin mouse monoclonal antibody (internal control) (1:5000, Cell Signaling Technology, 8H10D10)) overnight at 4 °C followed by the incubation of secondary antibodies (goat anti-rabbit IgG-HRP (1:10,000, Santa Cruz Biotechnology, sc-2004) for ACOT7, donkey anti-mouse IgG-HRP (1:10,000, Abcam, ab97030) for β-Actin).

### Quantification of metabolites of lipid metabolism

The differentiated mouse 3T3-L1 adipocyte cells expressed with *pCDH-fACOT7*, *pCDH-fACOT7mut*, *pCDH-fATGL*, *pCDH-fATGLmut* or *pCDH* were collected in a 1.5 ml Eppendorf tube. The internal control (Methyl tridecanoate) (Sigma) and 1 ml solution composing of 2% H_2_SO_4_ and 98% methanol (Sigma) were added to each tube followed by the incubation at 80 °C for one hour. Subsequently, 0.3 ml hexane (Sigma) and 1.5 ml H_2_O were added and mixed, followed by centrifuging at 5000 rpm for 10 min, after which the fatty acids were dissolved onto the hexane layer. The organic phase (hexane layer) was analyzed using coupled gas chromatography-mass spectrometry (Agilent). Compounds were identified by comparing their retention time and spectra with those of authentic reference compounds in the NIST 02 MS Libraries (Rev. D.04.00, Agilent). The mean comparison between different cell types was conducted by a paired t test after the normality test in *R*.

### Metabolic comparisons between *ATGL* and *ACOT7* mutations

The FFA differences caused by *ATGL* mutation (i.e. FFA abundance of wild type minus that of mutant) were compared to that caused by *ACOT7* mutation. The significance level was examined by an ANOVA test in *R*.

### *ATGL* and *ACOT7* gene expression in pectoral muscles

The pectoral muscle RNA-seq data of chickens (accession number: SRR7356426, SRR7356427, SRR7356428, SRR7356429, SRR7356430, SRR7356431) and zebra finches (SRR2545941, SRR2545942, SRR2545943, SRR2545947, SRR2545948, SRR2545949) were obtained from GenBank. The expression level of each gene was quantified using *RPKM* by mapping the RNA-seq reads onto the chicken and zebra finch (Ensembl89) gene sets respectively.

### Acyl-CoA and acetyl-CoA quantification in pectoral muscles

All the animal experiments were approved by the Institutional Animal Care and Use Committee of Institute of Zoology, Chinese Academy of Sciences. Pectoral muscles were collected from three flight-degenerate bird species (ostrich, chicken, turkey) and three flying bird species (zebra finch, budgerigar and domesticated pigeon) with *N* = 3 for each species. The extraction of acetyl-CoAs and long-chain acyl-CoAs from the muscles was carried out as previously described with some modifications^65^. Briefly, the sample was weighed in a 2 ml Sarstedt tube with 300 µL of extraction buffer containing isopropanol, 50 mM KH_2_PO_4_, 50 mg/mL BSA (25:25:1 v/v/v) acidified with the addition of glacial acetic acid. Next, 19:0-CoA was added as an internal standard and the tissues were homogenized on a bead ruptor (Omni) at the conditions optimized for muscles (8 m/s, 8 s, 2 cycles, pause = 5 s). Following homogenization, 300 µL of petroleum ether was added and the sample was centrifuged at 9000 rpm for 2 min at 4 °C. The upper phase was removed. The samples were extracted two more times with petroleum ether as described above. To the lower phase finally remaining, 5 µL of saturated ammonium sulfate was added followed by 600 µL of chloroform:methanol (1:2 v/v). The sample was then incubated on a thermomixer at 450 rpm for 20 min at 25 °C, followed by a centrifuge at 12,000 rpm for 5 min at 4 °C. Clean supernatant was transferred to fresh tube and subsequently dried in the SpeedVac under OH mode (Genevac). Dry extracts were resuspended in appropriate volume of methanol:water (9:1 v/v) prior to liquid chromatography–mass spectrometry (LC–MS) analyses (Agilent).

### Simulation of the functional changes of ATGL and ACOT7

To examine the effects of decreased lipid hydrolytic activity of ATGL and increased CoA thioester hydrolytic activity of ACOT7 on the lipid and carbohydrate metabolisms, we established a kinetic model based on the negative feedbacks between the two metabolisms^39^ defined as:

1 $$\frac{{dx}}{{dt}} = - \beta 1x + \frac{{\alpha 1}}{{1 + y^{n1}}}--\frac{{\alpha 3}}{{1 + y^{n3}}}$$

2 $$\frac{{dy}}{{dt}} = - \beta 2x + \frac{{\alpha 2}}{{1 + x^{n2}}}$$

where *x* is the concentration of acyl-CoA, *y* is the concentration of carbohydrate, *t* is the time series, *ß*1 and *ß*2 are the consumption rates of *x* and *y* for acyl-CoA and carbohydrate metabolisms respectively, *α*1 is the hydrolytic activity of ATGL catalyzing lipids to generate acyl-CoA, *α*2 is the hydrolitic activity of enzyme catalyzing carbohydrates to generate monosaccharides, *α*3 is the CoA thioester hydrolytic activity of ACOT7 hydrolyzing acyl-CoA, *n*1, *n*2, and *n*3 are Hill coefficients, indicating the inhibition efficiency of *y* to *x* and *x* to *y*, respectively. Larger values of *α*1, *α*3 indicate higher lipid hydrolytic activity of ATGL and higher hydrolytic activity of ACOT7, respectively.

When rescaled in unit of carbohydrate concentration, the ATP output (*z*) could be estimated as *z* = 2.25*x* + *y* (3), because lipids generate ~2.25 times of the energy as the same quantity of carbohydrates do^66^.

In our case, we set *ß*1 = *ß*2 = 1, *n*1 = *n*2 = *n*3 = 1, *α*2 = 5, *α*3 = 10. We decreased *α*1 from 25 to 10 to assess how the reduction of ATGL hydrolytic activity on lipids affects the metabolisms of lipids and carbohydrates. We also tried other values for *ß*1, *ß*2, *n*1, *n*2, *α*2 and they showed the similar patterns when decreasing *α*1 (not shown). It is noted that we kept *α*3 < *α*1 during the simulation because the effect of ATGL hydrolytic activity on lipids is larger than that of ACOT7, as stated in the main text.

To assess how the increase of hydrolytic activity of ACOT7 affects the metabolism of lipids and carbohydrates, we set *ß*1 = *ß*2 = 1, *n*1 = *n*2 = *n*3 = 1, *α*2 = 5, *α*1 = 10 and increased *α*3 from 1 to 10.

### Selection analysis

To identify whether convergent sites in *ATGL* and *ACOT7* are driven by natural selection, we carried out the positive selection analysis using the *codeml* implemented in PAML v4.7. For each convergent gene, multiple sequences were aligned using SATé-II^31^, and the alignment was input to *codeml*. The phylogenetic tree is obtained from the previous study^27^ we contributed to, with flight-degenerate bird species set as foreground and flying bird species as background. The branch-site model was implemented to identify the positive selection signature.

### Ancestral codon reconstruction for ATGL^321^ and ACOT7^197^

For the reconstruction, we used the phylogenetic tree of 48 avian species^5^ that we contributed to with *Alligator mississippiensis* as the outgroup (Fig. 6). The ancestral states of the two convergent loci at internal nodes of the studied avian phylogeny were inferred by the *codeml* program implemented in PAML with the settings recommended by a previous study^67^. To confirm the results, we selected 83 of 103 avian (species) genomes above mentioned (14 flight-degenerate and 69 flying species), in which both *ATGL* and *ACOT7* gene sequences were available. For the PAML reconstruction of the two focal sites among the 83 species, we used the three avian phylogenies recently published^27,68,69^ and 14 possible tree topologies newly generated, respectively. The 14 trees were generated through the random combination of four typically unresolved branches with low bootstrapping values (<80) in an avian phylogenetic tree^69^ previously reported (details see Supplementary Fig. 4a).

### Reporting summary

Further information on research design is available in the Nature Research Reporting Summary linked to this article.

## Supplementary information

Supplementary Information

Reporting Summary

Source Data

## Data Availability

Genomes of 103 bird species were downloaded from GenBank(URLs: http://gigadb.org/dataset/101000; https://www.ncbi.nlm.nih.gov/genome). A reporting summary for this article is available as a Supplementary Information file and the source data underlying Figs. 1b, c, e, f, 2a–c, 3, 4a–d, 5 and Supplementary Figs. 1–3 are provided as a Source Data file.

## Code availability

The scripts for the orthologous gene identification and convergent evolutionary analysis in this study have been deposited at github (https://github.com/Pussinboots123/Orthologous-identification).
