# Supplementary material for: Convergent genomic signatures of flight loss in birds suggest a switch of main fuel
Source: Nat Commun. 2019 Jun 21;10:2756. doi: 10.1038/s41467-019-10682-3 (PMC6588704; doi:10.1038/s41467-019-10682-3)
Supplement: Supplementary file 1 — Supplementary Information [file 41467_2019_10682_MOESM1_ESM.pdf]

Supplementary Information for

Convergent genomic signatures of flight loss in birds suggest a switch of main fuel

Pan et al.

Correspondence to: zhanxj@ioz.ac.cn

This PDF file includes:

Supplementary Figures 1-4

Supplementary Tables 1-3

Supplementary Figures

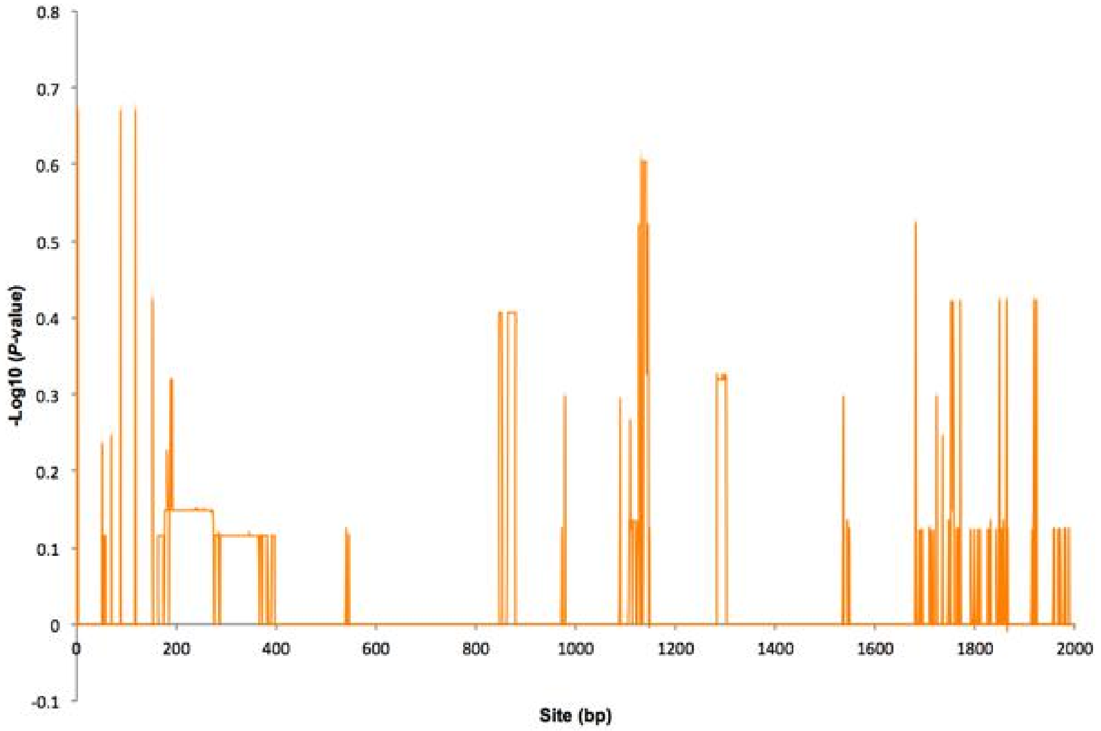

Supplementary Figure 1. The log-transformed *P* values for all sites on *CUX1*. *P* of each nucleotide is calculated based on its frequency difference between flying and flight-degenerate avian species using Fisher's exact test. It is noted that all *P* > 0.05 in the analysis. Source data are provided as a Source Data file.

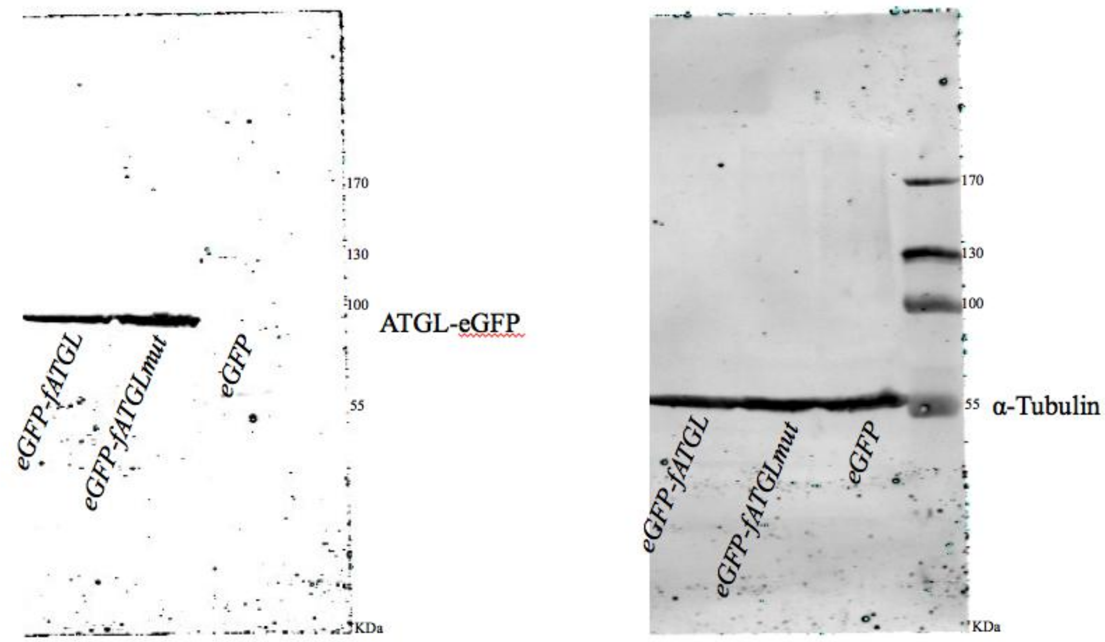

Supplementary Figure 2. Western blotting results for HeLa cells transfected with plasmids containing *ATGL* wild type (*eGFP-fATGL*), *ATGL* mutant type (*eGFP-fATGLmut*) and negative control (*eGFP*), respectively. α-Tubulin protein was used as the internal control. Source data are provided as a Source Data file.

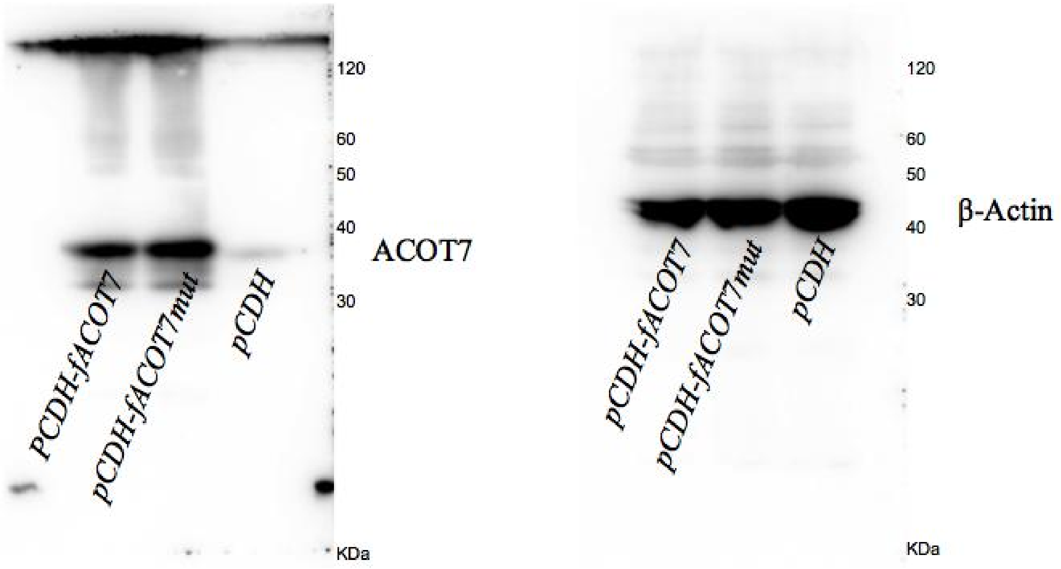

Supplementary Figure 3. Western blotting results for the differentiated 3T3-L1 cells expressed with wild type (*pCDH-fACOT7*), mutant type (*pCHD-fACOT7mut*) and negative control (*pCDH*), respectively. β-Actin protein was used as the internal control. Source data are provided as a Source Data file.

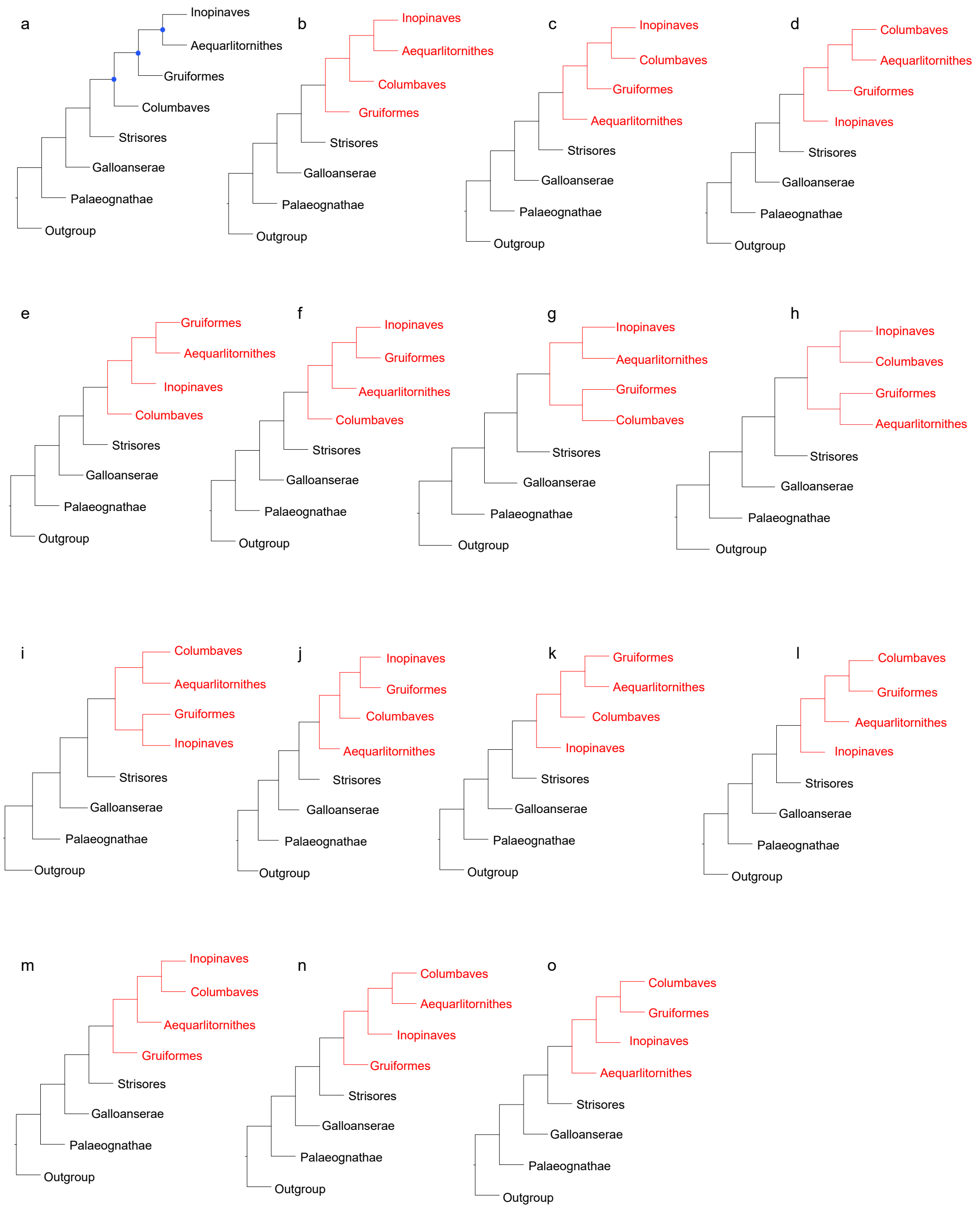

Supplementary Figure 4. The possible topologies derived from a previous avian phylogenetic tree[1] (**a**) containing three nodes with bootstrapping value less than 80 (blue dots). For each possible topology, we performed the ancestral state reconstruction of focal sites in the 83 studied avian species (Supplementary table 2) using the American alligator as the outgroup[2].

Supplementary Tables

Supplementary Table 1. Results of the two tests for 29 genes containing potential convergent nucleotides in eight flight-degenerate bird species.

Ensembl ID of chicken transcript	FDB-FB frequency ($P < 0.001$, Fisher's exact test)	Zou & Zhang's method[3] ($P < 0.01$, Poisson test)	Gene name
ENSGALP00000023449	+	+	*Adipose triglyceride lipase*
GALT00000000926	+	+	*Acyl-CoA thioesterase 7*
GALT00000027716	+	+	*Nuclear protein, ataxia-telangiectasia locus*
ENSGALT00000015426	+	-	*Alpha-actinin 1*
ENSGALT00000015253	+	-	*Lanosterol 14-alpha demethylase*
ENSGALT00000017668	+	-	*Histone H2A deubiquitinase*
ENSGALT00000020547	+	-	*E3 ubiquitin-protein ligase*
ENSGALT00000022129	+	-	*Pericentriolar material 1 protein-like*
ENSGALT00000025680	+	-	*PAX3- and PAX7-binding protein 1*
ENSGALT00000036681	+	+	*XK-related protein 9*
ENSGALT00000037442	+	-	*Zinc finger and BTB domain-containing protein 18*
ENSGALT00000003326	+	-	*WD repeat-containing protein 47*
GALT00000001466	+	-	*GTPase-activating protein and VPS9 domain-containing protein 1-like*
ENSGALT00000003701	+	-	*PMS1 protein homolog 1*
GALT00000005337	+	-	*SUMO-interacting motif-containing protein 1*
GALT00000008827	+	-	*Interleukin-17 receptor D precursor*
GALT00000009107	+	-	*Filamin-B*
GALT00000011624	+	-	*Probable cation-transporting ATPase 13A4*
GALT00000012220	+	-	*Run domain Beclin-1-interacting and cysteine-rich domain-containing protein*
GALT00000015011	+	-	*Tetratricopeptide repeat protein 30A*
GALT00000015779	+	+	*Inactive carboxypeptidase-like protein*
GALT00000016989	+	-	*Fatty-acid amide hydrolase 1*
GALT00000017201	+	-	*HEAT repeat-containing protein 5B*
GALT00000019412	+	+	*Cytochrome P450*
GALT00000023908	+	+	*Basic helix-loop-helix domain-containing protein*
GALT00000026601	+	-	*Apolipoprotein B precursor*

GALT00000033133	+	-	*Gamma-adducin*
GALT00000034685	+	+	*HDAC3 splicing HDAC3beta*
ENSGALT00000008427	+	-	*Sphingosine-1-phosphate phosphatase 2-like*

Note: FDB means flight-degenerate bird species and FB flying bird species.

Supplementary Table 2. Codons of *ATGL*^{321} and *ACOT7*^{197} in 103 avian species from GenBank.

Species	English name	*ATGL*	*ACOT7*
Pseudopodoces humilis	Hume's groundpecker	*AAT*	*GCG*
Aquila chrysaetos canadensis	Golden eagle	*AGT*	*GCC*
Zonotrichia albicollis	White-throated Sparrow	*AGT*	*GCG*
Serinus canaria	Canary	*AGT*	*GCG*
Agapornis roseicollis	Rosy-faced lovebird	*AGT*	*GCA*
Amazona vittata	Puerto rican parrot	*AGT*	*GCC*
Ara macao	Scarlet macaw	*AGT*	*GCA*
Amazona aestiva	Turquoise-fronted amazon	*AGT*	*GCC*
Phylloscopus plumbeitarsus	Two-barred warbler	*AGT*	*GCG*
Passer domesticus	House sparrow	*AGT*	*GCG*
Setophaga coronata coronata	Myrtle warbler	*AGT*	*GCG*
Ciconia boyciana	Oriental white stork	*AGT*	*GCG*
Grus japonensis	Red-crowned crane	*AGT*	*GCG*
Patagioenas fasciata monilis	Band-tailed pigeon	*AGC*	*GCG*
Sporophila hypoxantha	Tawny-bellied seedeater	*AGT*	*GCG*
Urile pelagicus	Sea cormorant	*AGT*	-
Nannopterum auritus	Double-crested cormorant	*AGT*	-
Nannopterum brasilianus	Neotropical cormorant	*AGT*	-
Uria lomvia	Thick-billed murre	*AGT*	*GCG*
Phylloscopus trochilus acredula	Willow warbler	*AGT*	*GCG*
Strix occidentalis caurina	Northern spotted owl	*AGT*	*GCG*
Calidris pugnax	Ruff	*AGT*	*GCG*
Sturnus vulgaris	Common starling	*AGT*	*GCG*
Parus major	Great tit	*AAT*	*GCG*
Lepidothrix coronata	Blue-crowned manakin	*AGT*	-
Phylloscopus trochiloides viridanus	Greenish warbler	*AGT*	*GCG*
Saxicola maurus maurus	Siberian stonechat	*AGT*	*GCG*
Zosterops lateralis melanops	Silvereye	*AGT*	*GCG*
Ficedula albicollis	Collared flycatcher	*AGT*	*GCG*
*Apaloderma vittatum**	Bar-tailed trogon	*GGT*	*GCG*
Anas zonorhyncha	Spot-billed duck	*GGT*	*GCG*
Anser brachyrhynchus	Pink-footed goose	*GGT*	*GCG*
Anser cygnoides	Swan goose	*GGT*	*GCG*
*Anas platyrhynchos**	Domesticated duck	*GGT*	*GCG*
*Aptenodytes forsteri**	Emperor penguin	*AGT*	-
*Chlamydotis macqueenii**	MacQueen's bustard	*AGT*	-
*Balearica regulorum gibbericeps**	Crowned crane	*AGT*	*GCG*
*Buceros rhinoceros silvestris**	Javan rhinoceros hornbill	*AGT*	*GCA*
*Cariama cristata**	Red-legged seriema	*AGT*	*GCG*

*Columba livia**	Pigeon	*AGC*	*GCG*
*Falco peregrinus**	Peregrine falcon	*AGT*	*GCG*
Falco cherrug	Saker falcon	*AGT*	*GCG*
*Fulmarus glacialis**	Northern fulmar	*AGT*	*GCG*
*Gavia stellata**	Red throated loon	*AGT*	*GCA*
*Merops nubicus**	Northern carmine bee-eater	-	*GCG*
*Nestor notabilis**	Kea	*AGT*	*GCG*
*Nipponia nippon**	Crested ibis	*AGT*	*GCG*
*Pelecanus crispus**	Dalmatian pelican	*AGT*	*GCG*
*Phaethon lepturus**	White-tailed tropicbird	*AGT*	*GCA*
*Phalacrocorax carbo**	Great black cormorant	*AGT*	-
*Phoenicopterus ruber**	Caribbean flamingo	*AGT*	*GCA*
*Podiceps cristatus**	Great-crested grebe	*AGT*	*GCA*
*Pterocles guturalis**	Yellow-thoated sandgrouse	*AGT*	*GCG*
*Pygoscelis adeliae**	Adelie penguin	*AGT*	-
*Tyto alba**	Barn owl	*AGT*	-
*Tauraco erythrolophus**	Angola turaco	*AGT*	*GCG*
*Chaetura pelagica**	Chimney swift	*AGT*	*GCG*
*Corvus brachyrhynchos**	American crow	*AGT*	*GCG*
*Mesitornis unicolor**	Brown mesite	*AGT*	*GCG*
*Egretta garzetta**	Little egret	*AGC*	*GCG*
*Melopsittacus undulatus**	Budgerigar	*AGT*	-
*Antrostomus carolinensis**	Chuck will's widow	*AGT*	*GCG*
*Haliaeetus albicilla**	White-tail eagle	*AGT*	*GCC*
*Leptosomus discolor**	Cuckoo roller	*AGT*	*GCG*
*Cathartes aura**	Turkey vulture	*AGT*	*GCG*
*Calypte anna**	Anna's hummingbird	*AGT*	*GCA*
*Geospiza fortis**	Medium ground finch	*AGT*	*GCG*
*Manacus vitellinus**	Golden-collared manakin	*AGT*	-
*Picoides pubescens**	Downy woodpecker	*AGC*	*GCG*
*Charadrius vociferus**	Killdeer	*AGT*	*GCG*
*Taeniopygia guttata**	Zebra finch	*AGT*	*GCG*
*Cuculus canorus**	Common cuckoo	*AGT*	*GCG*
*Haliaeetus leucocephalus**	Bald eagle	*AGT*	*GCC*
Athene cunicularia	Burrowing owl	*AGT*	*GCG*
Pygoscelis papua	Gentoo penguin	*AGT*	-
Psittacula krameri	Rose-ringed Parakeet	*AGT*	*GCA*
Pygoscelis antarcticus	Chinstrap penguin	*AGT*	-
Spheniscus mendiculus	Galapagos Penguin	*AGT*	-
Spheniscus humboldti	Humboldt penguin	*AGT*	-
Spheniscus magellanicus	Magellanic penguin	*AGT*	-
Nannopterum harrisi	Cormorant	*AGT*	*GCA*

Acridotheres javanicus	White-vented Myna	*AGT*	-
Cyanistes caeruleus	Eurasian Blue Tit	*AAT*	*GCG*
Empidonax traillii	Willow flycatcher	*AGT*	-
Hemignathus virens	Hawaii Amakihi	*AGT*	*GTG*
Lonchura striata	White-backed munia	*AGT*	*GCG*
Limosa lapponica	Black-tailed godwit	*AGT*	*GCG*
Apteryx australis mantelli	Brown kiwi	*GGG*	-
Colinus virginianus	Northern bobwhite	*GGT*	-
Bambusicola thoracicus	Chinese Bamboo Partridge	*GGT*	*GTG*
Callipepla squamata	Blue quail	*GGT*	*GTG*
Coturnix japonica	Japanese quail	*GGT*	*GTG*
Numida meleagris	Helmet guineafowl	*GGT*	*GTG*
Lyrurus tetrix tetrix	Black grouse	*GGT*	*GTG*
Tympanuchus cupido pinnatus	Greater prairie chicken	*GGT*	*GCG*
*Meleagris gallopavo**	Turkey	*GGT*	*GTG*
*Struthio camelus**	Ostrich	*GGG*	*GTG*
*Colius striatus**	Speckled mousebird	*GGT*	*GTG*
*Acanthisitta chloris**	Rifleman	*GGT*	*GTG*
*Eurypyga helias**	Sunbittern	*GGT*	*GTG*
*Opisthocomus hoazin**	Hoatzin	*GGT*	*GTG*
*Tinamus guttatus**	White throated tinamou	*GGG*	*GTG*
*Gallus gallus**	Chicken	*GGT*	*GTG*

Note: Flight-degenerate species were in red. *48 bird genomes that we used in the screening of convergent sites from the 15,239 orthologous genes. - missing in the genome assembly.

Supplementary Table 3. qPCR primers of the genes for the maturation determination of mouse preadipocytes

Gene name	Forward primer	Reverse primer
CEBPβ	ACGACTTCCTCTCCGACCTCT	CGAGGCTCACGTAACCGTAGT
CEBPα	CAAGAACAGCAACGAGTACCG	GTCACTGGTCAACTCCAGCAC
PPARγ	GCCCTTTGGTGACTTTATGG	CAGCAGGTTGTCTTGGATGT
GAPDH	GTATGACTCCACTCACGGCAAA	GGTCTCGCTCCTGGAAGATG
Cyclophilin A	CAAATGCTGGACCAAACACAA	GCCATCCAGCCATTCAGTCT

References

1. Prum, R. O. et al. A comprehensive phylogeny of birds (Aves) using targeted next-generation DNA sequencing. *Nature* **526**, 569-573 (2015).
2. Green, R. E. et al. Three crocodilian genomes reveal ancestral patterns of evolution among archosaurs. *Science* **346**, 1254449 (2014).
3. Zou, Z. & Zhang, J. Are convergent and parallel amino acid substitutions in protein evolution more prevalent than neutral expectations? *Mol. Biol. Evol.* **32**, 2085-2096 (2015).
