# Supplementary material for: Convergent genomic signatures of flight loss in birds suggest a switch of main fuel
Source: Nat Commun. 2019 Jun 21;10:2756. doi: 10.1038/s41467-019-10682-3 (PMC6588704; doi:10.1038/s41467-019-10682-3)
Supplement: Supplementary file 2 — Reporting Summary [file 41467_2019_10682_MOESM2_ESM.pdf]

nature research

Corresponding author(s): DBPR NCOMMS-18-26475A

Last updated by author(s): Feb 15, 2019

Reporting Summary

Nature Research wishes to improve the reproducibility of the work that we publish. This form provides structure for consistency and transparency in reporting. For further information on Nature Research policies, see Authors & Referees and the Editorial Policy Checklist.

Statistics

For all statistical analyses, confirm that the following items are present in the figure legend, table legend, main text, or Methods section.

n/a	Confirmed	
☐	☒	The exact sample size (*n*) for each experimental group/condition, given as a discrete number and unit of measurement
☐	☒	A statement on whether measurements were taken from distinct samples or whether the same sample was measured repeatedly
☐	☒	The statistical test(s) used AND whether they are one- or two-sided<br>*Only common tests should be described solely by name; describe more complex techniques in the Methods section.*
☒	☐	A description of all covariates tested
☐	☒	A description of any assumptions or corrections, such as tests of normality and adjustment for multiple comparisons
☐	☒	A full description of the statistical parameters including central tendency (e.g. means) or other basic estimates (e.g. regression coefficient) AND variation (e.g. standard deviation) or associated estimates of uncertainty (e.g. confidence intervals)
☐	☒	For null hypothesis testing, the test statistic (e.g. *F*, *t*, *r*) with confidence intervals, effect sizes, degrees of freedom and *P* value noted<br>*Give P values as exact values whenever suitable.*
☒	☐	For Bayesian analysis, information on the choice of priors and Markov chain Monte Carlo settings
☒	☐	For hierarchical and complex designs, identification of the appropriate level for tests and full reporting of outcomes
☒	☐	Estimates of effect sizes (e.g. Cohen's *d*, Pearson's *r*), indicating how they were calculated

Our web collection on statistics for biologists contains articles on many of the points above.

Software and code

Policy information about availability of computer code

Data collection	Data was directly downloaded from the link, http://phybirds.genomics.org.cn/download.jsp, Ensembl and GenBank. Therefore no software was used.
Data analysis	SATé-II, BLAST v2.2.26, PAML v4.76, R v3.3.1, Python v2.7, and ImageJ v1.51h. The scripts for the orthologous gene identification and convergent evolutionary analysis in this study have been deposited at github.

For manuscripts utilizing custom algorithms or software that are central to the research but not yet described in published literature, software must be made available to editors/reviewers. We strongly encourage code deposition in a community repository (e.g. GitHub). See the Nature Research guidelines for submitting code & software for further information.

Data

Policy information about availability of data

All manuscripts must include a data availability statement. This statement should provide the following information, where applicable:

- Accession codes, unique identifiers, or web links for publicly available datasets
- A list of figures that have associated raw data
- A description of any restrictions on data availability

All the raw genome data were publicly available from http://phybirds.genomics.org.cn/download.jsp, Ensembl and GenBank. The source data underlying Figs 1b, c, e, f, 2a-c, 3, 4a-d, 5 and Supplementary Figs 1-3 are provided as a Source Data file.

Field-specific reporting

Please select the one below that is the best fit for your research. If you are not sure, read the appropriate sections before making your selection.

☒ Life sciences ☐ Behavioural & social sciences ☐ Ecological, evolutionary & environmental sciences

For a reference copy of the document with all sections, see nature.com/documents/nr-reporting-summary-flat.pdf

Life sciences study design

All studies must disclose on these points even when the disclosure is negative.

Sample size	We have established a large database of 48 well-assembled avian genomes (e.g. Zhang et al. 2014). Furthermore, we have used additional 55 avian genomes to verify our conclusions of convergent evolution and ancestral state reconstruction. These are all the avian genomes that we could download from GenBank until 08/11/2018
Data exclusions	No data has been excluded.
Replication	All experiments are replicated at least three times and all of the results were provided in Figures.
Randomization	Randomization was not required to this study.
Blinding	Not applicable.

Reporting for specific materials, systems and methods

We require information from authors about some types of materials, experimental systems and methods used in many studies. Here, indicate whether each material, system or method listed is relevant to your study. If you are not sure if a list item applies to your research, read the appropriate section before selecting a response.

Materials & experimental systems

n/a	Involved in the study	
☐	☒	Antibodies
☐	☒	Eukaryotic cell lines
☒	☐	Palaeontology
☐	☒	Animals and other organisms
☒	☐	Human research participants
☒	☐	Clinical data

Methods

n/a	Involved in the study	
☒	☐	ChIP-seq
☒	☐	Flow cytometry
☒	☐	MRI-based neuroimaging

Antibodies

Antibodies used	ATGL antibody (Cell Signaling Technology, 2138S), α-Tubulin (Abcam, ab7291), goat anti-rabbit antibody (LI-COR Biosciences, 926-68071), donkey anti-mouse antibody (LI-COR Biosciences, 926-32212), ACOT7 rabbit polyclonal antibody (Proteintech, 15972-1-AP), β-Actin mouse monoclonal antibody (Cell Signaling Technology, 8H10D10), goat anti-rabbit IgG-HRP (Santa Cruz Biotechnology, sc-2004), and donkey anti-mouse IgG-HRP (Abcam, ab97030).
Validation	The ATGL antibody was obtained from Cell Signaling Technology and α-Tubulin from Abcam. The goat anti-rabbit antibody and donkey anti-mouse antibody were from LI-COR Biosciences. ACOT7 rabbit polyclonal antibody was from Proteintech and β-Actin mouse monoclonal antibody from Cell Signaling Technology. The goat anti-rabbit IgG-HRP was from Santa Cruz Biotechnology and the donkey anti-mouse IgG-HRP from Abcam.

Eukaryotic cell lines

Policy information about cell lines

Cell line source(s)	The human HeLa cells were obtained from American Type Culture Collection. The human embryonic kidney 293FT cells were obtained from National Infrastructure of Cell Line Resource. The mouse 3T3-L1 cells were obtained from Shanghai Institutes for Biological Sciences, Chinese Academy of Sciences, China.
Authentication	The human HeLa cells had been authenticated by American Type Culture Collection. The human embryonic kidney 293FT cells had been authenticated by National Infrastructure of Cell Line Resource. The mouse 3T3L1 cells had been authenticated by Shanghai Institutes for Biological Sciences, Chinese Academy of Sciences.

Mycoplasma contamination	The cells were not tested for Mycoplasma contamination.
Commonly misidentified lines (See ICLAC register)	None.

Animals and other organisms

Policy information about studies involving animals; ARRIVE guidelines recommended for reporting animal research

Laboratory animals	This study didn't involve laboratory animals.
Wild animals	Captive-bred zebra finches, pigeons, parrots, chickens, ostriches, and turkeys aged less than 1 year old (N = 3 for each species) were obtained from the farm, and the sexes were chosen randomly. All birds were sacrificed for obtaining flight muscles. All the animal experiments were approved by the Institutional Animal Care and Use Committee of Institute of Zoology, Chinese Academy of Sciences.
Field-collected samples	This study didn't involve field-collected samples.
Ethics oversight	All the animal experiments were approved by the Institutional Animal Care and Use Committee of Institute of Zoology, Chinese Academy of Sciences

Note that full information on the approval of the study protocol must also be provided in the manuscript.
